# Complete genomes of 21 avian pathogenic *Escherichia coli* isolated from colibacillosis cases in Georgia poultry

**DOI:** 10.1128/mra.00186-26

**Published:** 2026-05-14

**Authors:** Klao Runcharoon, Julia Ienes-Lima, Catherine M. Logue

**Affiliations:** 1 Department of Population Health, College of Veterinary Medicine, University of Georgia 1355, Athens, Georgia, USA; University of Maryland Baltimore, Baltimore, Maryland, USA

**Keywords:** Avian pathogenic *E. coli*, genomes, poultry, sequencing, colibacillosis, Georgia USA

## Abstract

Avian pathogenic *Escherichia coli* (APEC) causes colibacillosis in poultry, primarily from serogroups O1, O2, and O78. However, emerging APEC serogroups with unknown genomic characteristics were found. This study reports the complete genomes of 21 APEC strains isolated from colibacillosis cases in Georgia (GPEC), USA poultry.

## ANNOUNCEMENT

Avian pathogenic *E. coli* (APEC) causes colibacillosis, a significant global poultry health issue (1, 2). While complete genome sequences exist for common APEC serogroups (O1, O2, and O78) (3–5), others remain underexplored. Here, we report the complete genome sequences of 21 unusual APEC strains isolated from colibacillosis cases in Georgia poultry (6). All 21 APEC isolates were recovered from lesion swabs and plated on MacConkey agar at 37°C for 24 h (6). Suspect colonies were identified using 16S rRNA PCR (7) and stocked in Brain Heart Infusion (BHI) broth at −80°C in 20% glycerol.

For sequencing, a single colony from the first passage was cultured in BHI broth at 37°C for 24 h. Cell suspensions were centrifuged (ThermoFisher Scientific Sorvall Legend XTR) at 10,000×*g* for 2 min, and frozen pellets shipped to Mr. DNA (https://www.mrdnalab.com/, Shallowater, TX, USA) for whole genome sequencing.

Bacterial pellets were resuspended in 180 µL of ATL buffer (Qiagen, Germantown, MD), followed by genomic DNA extraction using the MagAttract high-molecular-weight DNA Kit (Qiagen). The DNA concentration was evaluated using Qubit dsDNA HS Assay Kit (Life Technologies, Carlsbad, CA). DNA quality was determined using a NanoDrop 2000 (ThermoFisher Scientific, Waltham, MA), followed by analysis using E-Gel SizeSelect 2% agarose gel (Invitrogen). Samples were sheared using the Covaris G-tube (Covaris Inc., Woburn, MA), and their average size was verified using the Agilent 2100 Bioanalyzer (Agilent Technologies, Alpharetta, GA). Libraries were prepared using the SMRTbell Express Template Prep Kit 2.0 (Pacific Biosciences, Menlo Park, CA), size selected >7 kb on the BluePippin instrument (Sage Science Inc., Beverly, MA), and sequenced using the PacBio Sequel IIe (Pacific Biosciences).

Genome assembly was performed using HIFIASM v0.24.0 (8). GPEC strains 1068 and 1258 were submitted to purge_dups v1.2.6 (9) to remove duplicate haplotypes identified during assembly. Assembled contigs were circularized in Geneious v2024 0.5 (10) and rotated to the *dnaA* gene using Circulator v1.5.5 (11). Assembly statistics, quality, and completeness were assessed with QUAST v5.2.0 (12) and BUSCO v5.8.3 (13) (see Table 1). All genomes were annotated using the NCBI Prokaryotic Annotation Pipeline v6.10 using the Best-placed reference protein set GeneMarkS-2+. Plasmid identification used PlasmidFinder 2.1 (14, 15). Serotypes were identified using the EcoH database, and virulence factors were identified using EcoliVF available on ABRicate v.1.0.1 (https://github.com/tseemann/abricate) with an ID threshold and minimum length of 85% and 60%, respectively. Default parameters were used for all software unless otherwise specified.

**TABLE 1:** Genomes and details of all sequenced strains^*a*^

APEC	Serotype	Genbank accession no.	SRA accession no.	Biosample accession no.	Total assembly length(bp)	Total reads (bp)	No. of contigs	N50	Chromosome size (bp)	CDS	GC content (%)	No. of plasmids
GPEC36	O17:H18	JBUBHW000000000	SRR37102318	SAMN54368641	5,146,629	91,750	3	4,930,328	4,930,328	4,923	50.70	2
GPEC102	O25:H4	JBUBJN000000000	SRR37102317	SAMN54368684	5,106,348	140,532	3	4,924,940	4,924,940	4,873	50.66	2
GPEC172	O25:H4	JBUBHX000000000	SRR37102305	SAMN54368642	5,327,067	82,420	4	5,026,865	5,026,865	5,140	50.6	3
GPEC548	Unidentified	JBUBIC000000000	SRR37102304	SAMN54368647	5,158,187	34,151	3	4,890,945	4,890,945	4,919	50.5	2
GPEC617	O86:H51	JBUBIE000000000	SRR37102303	SAMN54368649	5,054,859	36,504	3	4,833,568	4,833,568	4,848	50.6	2
GPEC709	O91:H7	JBUBII000000000	SRR37102306	SAMN54368653	5,084,985	31,327	2	4,878,742	4,878,742	4,835	50.4	1
GPEC755	O25:H4	JBUBIJ000000000	SRR37102302	SAMN54368654	5,133,893	37,188	3	4,904,457	4,904,457	4,926	50.6	2
GPEC874	O86:H2	JBUBIM000000000	SRR37102301	SAMN54368657	5,260,738	26,923	2	5,101,906	5,101,906	4,795	50.5	1
GPEC1031	O11:H4	JBUBIO000000000	SRR37102300	SAMN54368659	5,340,141	96,687	2	5,219,934	5,219,934	5,193	50.8	1
GPEC1042	O161:H4	JBUBIR000000000	SRR37102299	SAMN54368662	5,349,078	26,574	5	5,002,245	5,002,245	5,153	50.5	4
GPEC1068	O45:H19	JBUEGN000000000	SRR37106155	SAMN55060529	5,842,556	74,932	6	5,243,689	5,243,689	5,680	50.4	4
GPEC1165	O161:H4	JBUBIT000000000	SRR37102316	SAMN54368664	5,182,428	28,500	4	4,964,230	4,964,230	4,961	50.6	3
GPEC1189	O88:H25	JBUBIX000000000	SRR37102315	SAMN54368668	4,939,321	30,791	2	4,813,215	4,813,215	4,706	50.6	1
GPEC1201	O115:H20	JBUBIY000000000	SRR37102313	SAMN54368669	5,321,827	28,647	5	4,979,201	4,979,201	5,136	50.6	4
GPEC1240	O88:H8	JBUBJC000000000	SRR37102312	SAMN54368673	5,139,898	72,428	3	4,956,824	4,956,824	4,962	50.3	2
GPEC1258	O149:H4	JBUBJD000000000	SRR37102311	SAMN54368674	5,455,143	52,296	4	5,128,409	5,128,409	5,291	50.8	3
GPEC1292	O91:H34	JBUBJE000000000	SRR37102314	SAMN54368675	4,796,069	41,201	2	4,683,349	4,683,349	4,515	50.5	1
GPEC1352	Onovel12:H4	JBUBJG000000000	SRR37102310	SAMN54368677	5,314,483	28,730	5	5,029,638	5,029,638	5,121	50.6	4
GPEC1354	Onovel12:H4	JBUBJH000000000	SRR37102309	SAMN54368678	5,273,397	44,157	6	4,968,301	4,968,301	5,071	50.8	5
GPEC1366	O115:H9	JBUBJI000000000	SRR37102308	SAMN54368678	5,354,891	40,504	4	4,934,524	4,934,524	5,144	50.8	3
GPEC1446	O45:H9	JBUBJM000000000	SRR37102307	SAMN54368683	5,689,010	36,224	3	5,179,706	5,179,706	5,268	50.5	2

^*a*^ Runcharoon, Klao; Ienes-Lima, Julia; Logue, Catherine (2026). Table of Data detailing the genomes included in the Genome Announcement : Complete Genomes of 21 Avian Pathogenic *Escherichia coli *isolated from colibacillosis cases in Georgia poultry by Klao Runcharoon, Julia Ienes-Lima, and Catherine M. Logue. figshare. Dataset. https://doi.org/10.6084/m9.figshare.32077146.

One isolate, GPEC 1446 (O45:H9), harbors *eae* and *hly* genes associated with intimin and hemolysin (16), contains two plasmids, and harbors *bla_CTX-M-55_* and *bla_TEM_* genes, suggesting extended-spectrum β-lactamase (ESBL) resistance.

Three strains identified as O25b:H4:ST131 (GPEC 102, GPEC 172, and GPEC 755), a serotype linked with human urinary tract infections and ESBL resistance (17), showed survival in chicken serum and human urine (18).

Two strains were identified as Onovel12:H4 (GPEC 1352 and GPEC 1354), a serotype previously reported in broiler barns (19).

The genomic information of these 21 APEC isolates (Table 1) provides valuable data, enhancing the APEC genomic toolbox for future comparative genomic studies.

## Data Availability

The genome sequences of the 21 APEC strains were deposited in GenBank under the accession numbers shown in Table 1.
